# A comparative study of the impacts of unbalanced sample sizes on the four synthesized methods of meta-analytic structural equation modeling

**DOI:** 10.1186/s13104-017-2768-5

**Published:** 2017-09-06

**Authors:** Marzieh Alamolhoda, Seyyed Mohammad Taghi Ayatollahi, Zahra Bagheri

**Affiliations:** Department of Biostatistics, School of Medicine, Shiraz University of Medical Sciences, Shiraz, Iran

**Keywords:** Meta-analysis, Structural equation modeling, Unbalanced sample sizes, Combining correlation matrices

## Abstract

**Background:**

In the first stage of meta-analytic structural equation modeling (MASEM), researchers synthesized studies using univariate meta-analysis (UM) and multivariate meta-analysis (MM) approaches. The MM approaches are known to be of better performance than the UM approaches in the meta-analysis with equal sized studies. However in real situations, where the studies might be of different sizes, the empirical performance of these approaches is yet to be studied in the first and second stages of MASEM. The present study aimed to evaluate the performance of the UM and MM methods, having unequal sample sizes in different primary studies. Testing the homogeneity of correlation matrices and the empirical power, estimating the pooled correlation matrix and also, estimating parameters of a path model were investigated using these approaches by simulation.

**Results:**

The results of the first stage showed that Type I error rate was well under control at 0.05 level when the average sample sizes were 200 or more, irrespective of the types of the methods or the sample sizes used. Moreover, the relative percentage biases of the pooled correlation matrices were also lower than 2.5% for all methods. There was a dramatic decrease in the empirical power for all synthesis methods when the inequality of the sample sizes was increased. In fitting the path model at the second stage, MM methods provided better estimation of the parameters.

**Conclusions:**

This study showed the different performance of the four methods in the statistical power, especially when the sample sizes of primary studies were highly unequal. Moreover, in fitting the path model, the MM approaches provided better estimation of the parameters.

**Electronic supplementary material:**

The online version of this article (doi:10.1186/s13104-017-2768-5) contains supplementary material, which is available to authorized users.

## Background

Meta-analysis (MA), as a popular statistical technique, is used for the purpose of integrating and summarizing the findings of different studies in order to yield more precise and reliable effect size of interest across independent studies. The dramatic growth of structural equation modeling (SEM) techniques in different types of sciences has attracted the attention of researchers on the methods that utilized the ideas of MA and SEM in synthesizing the results of several studies [1]. The term meta-analytic structural equation modeling (MASEM) refers to a set of statistical techniques used for testing hypothetical models in psychology, medicine and management and accounting researches [2–4]. Two stages are considered when analyzing data in MASEM: the first stage involves a combination of correlation matrices of independent studies together to form a pooled correlation matrix, if the homogeneity hypothesis is held across studies. In the second stage, SEM analysis is performed to fit the SEM model by the pooled correlation matrix [1].

There are different methods for synthesizing correlation matrices in the first stage of MASEM. These methods are categorized as UM and MM methods. The UM methods are frequently used in applied researches [5–8]. Univariate-z (UNIz) and Univariate-r (UNIr), introduced by Hedges and Olkin [9] and Hunter and Schmidt [10], are the most popularly used UM techniques in MASEM researches. These approaches synthesize correlation matrices among ***k*** studies by taking the weighted average of correlation, $r_{i}$. However, one problem associated with these approaches is that they fail to take into account the dependencies between correlations. This can cause a bias estimation of the pooled correlation matrix [11]. Given this deficiency, MM methods have been proposed and applied to provide more accurate results. GLS and TSSEM are the two best MM methods introduced by Becker [12] and Cheung and Chan [13]. Becker used generalized least squares estimation method to model the dependency between correlation coefficients in the first stage. However, due to some poor performance of this method in comparison with UMs [13–15], the researchers recommended different modifications in order to improve the traditional GLS method [11, 14, 15]. In TSSEM approach, correlations are pooled by multiple group SEM techniques at stage one and the pooled matrix is used for the analysis of SEM in the second stage.

Previous studies have shown that MM approaches perform better than UMs and also provide results with good and relatively unbiased estimators [13–17]. It should be noted that in most of the previous studies, the comparison between the mentioned methods and their properties was based on equal sample sizes within each MA. However, usually this does not occur in actual practice. Since prior results showed that trial sample sizes, *n*, influence treatment effect estimates substantially [18], it was hypothesized that these methods would perform inadequately, if a combination of very unequal-sized studies are included in an MA. Such a situation is not uncommon and frequently occurs, especially in clinical trials and medical sciences. For example, in the sample of 22,453 meta-analyses, Davey et al. demonstrated that in general, the sample size of individual studies varied considerably across MAs with a median of 91, an interquartile range from 44 to 210 and maximum of 1,242,071 individuals. They also concluded that sample sizes varied substantially across medical specialties, with the lowest and highest values of median size (61 and 154) for pathological conditions, symptoms and signs and for cancer, respectively [19].

Although several simulation studies were carried out to compare the performance of the UM and MM approaches [11, 13, 17], there exist no empirical study to evaluate these methods when there is a mixture of very unequal sample sizes design in MA. Differences in the sample sizes of primary studies within each MA are one of the problems encountered by MA studies when dealing with meta-analytical methods [20]. To the best of our knowledge, comparisons between the methods with unequal sample sizes have been evaluated only in some studies in which the variation of sample size was obtained under the specific requirements of the formula and spatial distributions [15, 16, 21, 22]. Although the use of these uneven sample sizes for MA studies might improve the findings [22], the produced sample sizes did not have significant difference when compared with the equal sized studies.

This study aimed to assess the effect of different unequal sample sizes scenarios on the statistical properties of approaches and made comparison with equal sample sizes.

## Methods

### Study design

#### Homogeneous studies

**Fig. 1:**
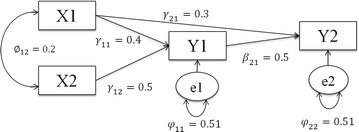
Path model used to simulate data samples.* X*_1_ and* X*_2_ are independent variables;* Y*_1_ is mediator;* Y*_2_ is dependent variable

A simulation study was conducted to evaluate the performance of UNIr, UNIz, MGLS and TSSEM approaches in both stages under different combinations of sample sizes. In this study, a path model with four observed variables was considered as shown in Fig. 1, which was already used by the pioneer researchers [17, 23].

The general form of the model is written as:

$$\varvec{Y} = \varvec{\varGamma X} + \varvec{BY} +\varvec{\zeta}$$

where $\varvec{ Y}_{2 \times 1}$ and $\varvec{X}_{2 \times 1}$ are vectors of endogenous and exogenous variables with $\varvec{B}_{2 \times 2}$ and $\varvec{\varGamma}_{2 \times 2}$ as their coefficients matrices, respectively. The term $\varvec{\zeta}_{2 \times 1}$ is the disturbance vector with variance–covariance matrix ${{\Psi }}_{2 \times 2}$. This model is an over-identified model with one degree of freedom. Population covariance matrix (${\Sigma }$) which is a function of the parameters model is given as:

$$\varvec{\varSigma}= \left[ {\begin{array}{*{20}c} {\left( {\varvec{I} - \varvec{B}} \right)^{ - 1} \left( {\varvec{\varGamma \varPhi \varGamma }^{\varvec{'}} +\varvec{\varPsi}} \right)\left( {\varvec{I} - \varvec{B}} \right)^{{\varvec{'} - 1}} } & {\left( {\varvec{I} - \varvec{B}} \right)^{ - 1} \varvec{\varGamma \varPhi }} \\ {\varvec{\varPhi \varGamma }^{\varvec{'}} \left( {\varvec{I} - \varvec{B}} \right)^{ - 1} } &\varvec{\varPhi}\\ \end{array} } \right],$$

where $\varvec{I}_{2 \times 2}$ and ${\varvec{\Phi}}_{2 \times 2}$ are identity matrix and covariance matrix of ***X***. If the model parameters are chosen as $${\varvec{\Gamma}} = \left[ {\begin{array}{*{20}c} {0.4} & {0.5} \\ {0.3} & 0 \\ \end{array} } \right]$$, $$\varvec{B} = \left[ {\begin{array}{*{20}c} 0 & 0 \\ {0.5} & 0 \\ \end{array} } \right]$$, $${\varvec{\Phi}} = \left[ {\begin{array}{*{20}c} 1 & {0.2} \\ {0.2} & 1 \\ \end{array} } \right]$$ and $${\Psi} = \left[ {\begin{array}{*{20}c} {0.51} & 0 \\ 0 & {0.51} \\ \end{array} } \right],$$ then the population covariance ($\varSigma$) implied by this model is derived as:

$$\varSigma = \left[ {\begin{array}{*{20}c} 1 & {} & {} & {} \\ {0.65} & 1 & {} & {} \\ {0.50} & {0.55} & 1 & {} \\ {0.58} & {0.35} & {0.20} & 1 \\ \end{array} } \right]$$

${\Sigma}$ can also serve as the common population correlation matrix. It was used to generate the simulated data. SEM techniques were used to estimate the parameters of the model [24].

#### Heterogeneous studies

In order to evaluate the statistical power of the four methods for rejecting homogeneity hypothesis correctly, another simulation study was performed in which simulated correlation matrices were classified into two homogeneous subgroups. Two fixed population matrices were used to represent between group differences under the fixed-effects model [13]. ${\Sigma}$ and ${\Sigma^{\prime}}$ were also used as two population correlation matrices under the fixed-effects model in order to generate the heterogeneous studies. Heterogeneity was assessed at two levels: 20% for small heterogeneity and 50% for large heterogeneity. This implied that 20, 50% of the correlation matrices were selected from another population matrix. Selection of the parameters of the path model was in such a way that the ${\Sigma^{\prime}}$ was obtained as:

$$\Sigma ^{{\prime }} = \left[ {\begin{array}{*{20}c} 1 & {} & {} & {} \\ {0.45} & 1 & {} & {} \\ {0.30} & {0.35} & 1 & {} \\ {0.40} & {0.19} & {0.15} & 1 \\ \end{array} } \right]$$

### Sample sizes

In MA of homogeneous and heterogeneous studies, the simulated data were based on three forms of the sample sizes designs: equal, moderately unequal and highly unequal sample sizes, such that the total sample size is the same. First, equal numbers of subjects were assigned to each MA studies. Second, for moderately unequal samples, the percentage of allocation of total sample sizes was considered as 40 and 60% for the large and small studies, respectively. At this point, larger studies had about 2.7 times more subjects than the small studies. Third, for highly unequal sized studies, the total sample size was assigned very unequally such that 40, 20 and 40% of the samples were selected as small, medium and large, respectively. In this case, studies with larger sample sizes had 1.6 and 4 times more subjects than the studies with medium and small sample sizes. For example, in the MA with five studies and $\bar{n} = 50$, the authors determined the sample sizes as 30, 30, 30, 80 and 80 for moderately unequal samples and 20, 20, 50, 80 and 80 for highly unequal samples.

The effects of inequality in each MA study and different values of number of studies (*k* = 5, 10 and 15) on the statistical properties of the four approaches and also the influence of heterogeneities on the statistical power of the four methods were also evaluated. A total of 1000 random samples were generated from multivariate normal distribution with a mean vector of zero and variance covariance matrix of ${\Sigma}$ in each simulation in order to achieve simulated correlation matrices. Moreover, the value of $\bar{n}$ per study was set at 50, 100, 200, 500 and 1000 subjects. Hence, this study included 15 MAs for each of the synthesizing methods.

### Estimation methods

In order to test the homogeneity of correlation matrices for the UM methods, the Bonferroni-adjusted at-least-one (BA1) approach [15] was used in the first stage. $Q_{GLS}$ and maximum likelihood (ML) methods which have been described by Cheung et al. [13] were used for the MGLS and TSSEM approaches, respectively. Rejection rates were calculated based on α = 0.05 in the first stage.

In the second stage, ML and asymptotically distributions free (ADF) estimation methods were used for fitting path model with UM and MM approaches, respectively. In addition, the total sample sizes were considered for the estimation of the parameters. For every parameter estimates, the relative percentage bias was defined as ${\text{Bias}}\left( {\hat{\theta }} \right) = \frac{{\bar{\hat{\theta }} - \theta }}{\theta } \times 100{\text{\% }}$. The value of $\bar{\hat{\theta }}$ is the mean of the estimates of the parameters in 1000 simulations and $\theta$ is the population value of the parameters.

The relative percentage bias of the standard error of each parameter estimate was used to assess the accuracy of the standard error estimates in fitting SEM. This value is defined as ${\text{Bias}}\,({\overline{SE}} ({\hat{{\theta }}})) = \frac{{\overline{SE} ({\hat{\theta }}) - SD({\hat{\theta }})}}{{SD({\hat{\theta }})}} \times 100{\text{\% }}$, where $\overline{SE} (\widehat{\varvec{\theta}})$ is the mean of the estimated standard errors and $SD(\hat{\varvec{\theta }})$ is the empirical standard deviation of the parameter estimates across 1000 replications. The values of less than 5% for the parameter estimates and 10% for the standard errors were treated as acceptable bias [25]. The R software version 3.2.1 was used to perform these simulation analyses using lavaan and metaSEM packages [26, 27]. The metaSEM runs under the OpenMx package [28].

## Results

### Results of stage 1

The results of observed rejection percentages of the present approaches for the simulated combinations of sample sizes in the first stage are shown in Table 1. With small average sample sizes (e.g., 50 and 100), there was over-rejection of the true model in some cases by UNIr, MGLS and TSSEM approaches. This over-rejection increased especially for TSSEM, when the number of studies and inequality in samples increased. However, UNIz approach performed very well under different sample sizes. The present findings revealed that the error rates were well under control under large sample sizes (e.g. 200 and above), regardless of the methods or the design of the sample sizes used for the analysis.

**Table 1:** Type I error rates of the methods for different combination of sample sizes in homogenous studies

$\bar{n}$	Types	k = 5				k = 10				k = 15			
		UNIr	UNIz	MGLS	TSSEM	UNIr	UNIz	MGLS	TSSEM	UNIr	UNIz	MGLS	TSSEM
50	E	4.4	3.8	4.8	6.7*	6.4*	5.4	6.8*	9.1*	6.7*	5.3	7.9*	10.7*
	M	5.6	4.2	5.7	6.7*	7.0*	5.2	7.2*	11.4*	8.1*	4.8	8.8*	11.0*
	H	6.7*	5.6	8.5*	10.0*	6.6*	4.6	8.6*	10.1*	8.5*	5.4	9.8*	13.3*
100	E	5.1	5.8	5.9	7.3*	5.2	5.3	5.2	6.7*	5.2	3.7	5.3	6.2
	M	5.9	5.2	6.0	7.1*	6.6*	4.3	6.2	7.4*	4.7	4.2	6.5*	6.6*
	H	5.9	4.3	6.0	5.7	6.2	4.7	6.7*	6.6*	6.8*	4.4	7.6*	8.3*
200	E	4.4	4.4	5.1	5.2	5.3	4.9	5.5	6.0	5.1	3.8	5.4	5.9
	M	4.5	3.3*	4.4	4.5	4.7	4.5	6.2	7.2*	4.9	4.1	5.1	5.6
	H	5.0	4.9	5.7	5.6	4.4	4.2	5.4	5.5	7.1*	6.0	6.5*	6.2
500	E	5.3	5.4	5.7	5.7	5.2	6.2	5.8	5.9	3.7	3.2*	5.3	5.5
	M	4.7	4.8	5.6	5.3	5.3	5.2	5.0	5.0	3.9	3.8	5.7	5.3
	H	5.4	4.7	4.9	5.2	5.1	4.3	5.0	5.3	5.2	4.5	6.4*	6.3
1000	E	4.8	5.0	5.0	5.4	4.9	5.3	4.2	4.2	4.2	4.2	5.3	5.3
	M	4.2	3.9	5.5	5.6	4.5	4.2	4.5	4.8	5.2	5.0	5.2	5.4
	H	4.7	5.0	5.7	5.6	4.3	4.6	5.2	5.6	4.1	3.9	4.5	5.2

$\bar{n}$ average sample sizes k number of studies, *UNIr* univariate-r, *UNIz* univariate-z, *MGLS* modified generalized least squared, *TSSEM* two-stage structural equation modeling, *E* equally sized studies *M* moderately unequal sized studies H highly unequal sized studies

* Values falls outside the 95% acceptance regions

Table 2 shows relative percentage biases of correlation coefficients obtained by four approaches at stage one. By comparing values with 2.5% which is known as an acceptable criterion [29], all the methods exhibited relative biases lower than 2.5% for all types of the sample sizes design. The values of relative percentage biases were approximately decreased with increasing average sample sizes, in almost all conditions. Furthermore, the findings showed that the UNIr and MGLS had the same relative percentage biases in almost all conditions.

**Table 2:** Relative percentage bias of correlation coefficients in the pooled correlation matrix at stage 1

Types	Average	UNIr						UNIz					
		*ρ*_21_	*ρ*_31_	*ρ*_41_	*ρ*_32_	*ρ*_42_	*ρ*_43_	*ρ*_21_	*ρ*_31_	*ρ*_41_	*ρ*_32_	*ρ*_42_	*ρ*_43_
k = 5													
50	E	−0.26	−0.37	−0.81	−0.99	−0.71	−0.46	0.68	0.84	0.31	0.13	0.73	1.10
	M	−0.40	−1.08	−0.41	−1.06	−0.76	−0.78	0.57	0.14	0.70	0.08	0.66	0.78
	H	−0.54	−0.85	−0.52	−1.04	−0.23	0.27	0.43	0.35	0.59	0.16	1.18	1.83
100	E	−0.43	−0.55	−0.39	−0.41	−0.34	−0.92	0.04	0.08	0.18	0.17	0.40	−0.15
	M	−0.19	−0.64	−0.17	−0.54	0.12	−0.02	0.27	−0.01	0.39	0.02	0.86	0.77
	H	−0.30	−0.39	−0.42	−0.57	−0.54	−0.18	0.17	0.21	0.14	−0.01	0.16	0.60
200	E	−0.12	0.15	−0.07	0.15	−0.05	0.70	0.10	0.45	0.18	0.44	0.29	1.08
	M	−0.14	−0.05	−0.23	−0.05	−0.28	−0.65	0.10	0.25	0.04	0.24	0.07	−0.26
	H	−0.03	0.00	−0.11	−0.07	−0.02	0.36	0.20	0.30	0.15	0.21	0.32	0.74
500	E	−0.09	0.02	−0.04	−0.07	−0.17	0.32	0.00	0.13	0.07	0.04	0.04	−0.03
	M	−0.05	−0.05	−0.16	−0.02	−0.02	0.08	0.04	0.07	−0.04	0.09	0.13	0.24
	H	−0.11	−0.08	−0.11	−0.11	−0.06	−0.01	−0.02	0.02	0.00	0.00	0.08	0.14
1000	E	−0.08	−0.13	0.01	−0.12	0.09	0.07	−0.04	−0.07	0.06	−0.07	0.16	0.15
	M	0.00	−0.10	−0.07	0.10	0.02	−0.11	0.04	−0.04	−0.02	0.15	0.09	−0.03
	H	−0.07	−0.03	−0.05	−0.04	−0.11	0.10	−0.02	0.03	0.01	0.01	−0.04	0.18
k = 10													
50	E	−0.57	−0.81	−0.34	−0.56	0.05	0.32	0.52	0.59	0.90	0.73	1.73	1.48
	M	−0.71	−0.56	−0.85	−0.77	−1.38	−0.60	0.39	0.86	0.37	0.54	0.25	1.22
	H	−0.51	−0.57	−0.64	−0.59	−0.79	−0.24	0.54	0.80	0.59	0.73	0.82	1.49
100	E	−0.39	−0.50	−0.33	−0.57	−0.94	−0.74	0.15	0.17	0.27	0.07	−0.14	0.11
	M	−0.52	−0.51	−0.43	−0.38	−0.81	−0.24	0.01	0.18	0.18	0.26	−0.02	0.62
	H	−0.23	−0.35	−0.21	−0.41	−0.36	−0.86	0.30	0.34	0.41	0.23	0.43	0.03
200	E	−0.16	−0.14	−0.16	−0.17	−0.05	0.20	0.10	0.20	0.14	0.15	0.35	0.64
	M	−0.13	−0.49	−0.18	−0.29	−0.32	−0.85	0.13	−0.15	0.11	0.03	0.08	−0.41
	H	−0.15	−0.39	−0.28	−0.22	−0.40	−0.48	0.11	−0.05	0.01	0.09	−0.01	−0.06
500	E	−0.06	−0.01	−0.06	−0.02	−0.16	−0.12	0.04	0.13	0.06	0.11	0.00	0.05
	M	−0.02	−0.08	−0.09	−0.18	−0.01	−0.26	0.09	0.06	0.03	−0.05	0.15	−0.09
	H	−0.09	−0.15	−0.08	−0.15	−0.14	−0.38	0.02	−0.01	0.04	−0.03	0.02	−0.21
1000	E	−0.02	−0.03	0.02	−0.02	0.09	0.31	0.03	0.04	0.08	0.04	0.17	0.40
	M	0.02	0.05	−0.03	0.03	0.06	0.11	0.08	0.12	0.03	0.09	0.14	0.19
	H	−0.03	−0.02	−0.06	−0.07	−0.05	−0.28	0.02	0.05	0.00	−0.01	0.03	−0.19
k = 15													
50	E	−0.61	−0.69	−0.57	−0.84	−1.04	−1.82	0.51	0.79	0.70	0.49	0.69	0.03
	M	−0.78	−0.84	−0.73	−1.14	−0.90	−1.45	0.33	0.60	0.57	0.22	0.76	0.37
	H	−0.64	−0.66	−0.70	−0.43	−1.21	−1.03	0.50	0.79	0.57	0.92	0.47	0.79
100	E	−0.17	−0.17	−0.30	−0.15	−0.37	0.20	0.38	0.54	0.32	0.51	0.46	1.12
	M	−0.31	−0.33	−0.26	−0.46	−0.44	−0.33	0.24	0.38	0.36	0.20	0.40	0.59
	H	−0.21	−0.13	−0.37	−0.28	−0.17	0.55	0.34	0.60	0.26	0.37	0.67	1.47
200	E	−0.14	−0.20	−0.21	−0.25	−0.11	−0.35	0.13	0.15	0.10	0.06	0.30	0.10
	M	−0.18	−0.27	−0.16	−0.18	−0.32	−0.51	0.09	0.09	0.15	0.15	0.10	−0.07
	H	−0.09	−0.07	−0.15	−0.17	−0.03	0.31	0.18	0.28	0.16	0.16	0.38	0.76
500	E	−0.11	−0.21	−0.16	0.13	−0.18	−0.39	−0.01	−0.07	−0.04	0.00	−0.02	−0.21
	M	−0.10	−0.20	−0.09	−0.20	0.05	−0.28	0.01	−0.06	0.04	−0.07	0.21	−0.10
	H	−0.06	−0.03	−0.02	−0.10	−0.04	0.04	0.05	0.11	0.10	0.03	0.12	0.21
1000	E	−0.01	−0.05	−0.08	−0.05	−0.11	−0.18	0.05	0.03	−0.02	0.03	−0.03	−0.09
	M	−0.02	−0.04	−0.03	−0.02	−0.09	−0.06	0.04	0.03	0.04	0.05	−0.01	0.03
	H	−0.02	−0.03	−0.02	−0.07	−0.06	−0.10	0.03	0.04	0.05	0.00	0.02	−0.01

Types	Average	MGLS						TSSEM					
		*ρ*_21_	*ρ*_31_	*ρ*_41_	*ρ*_32_	*ρ*_42_	*ρ*_43_	*ρ*_21_	*ρ*_31_	*ρ*_41_	*ρ*_32_	*ρ*_42_	*ρ*_43_
k = 5													
50	E	−0.26	−0.37	−0.81	−0.99	−0.71	−0.46	0.64	0.75	0.24	0.10	0.63	1.00
	M	−0.40	−1.07	−0.40	−1.05	−0.75	−0.77	0.52	0.04	0.63	0.05	0.56	0.57
	H	−0.54	−0.85	−0.51	−1.04	−0.23	0.28	0.40	0.31	0.53	0.14	1.08	1.69
100	E	−0.43	−0.55	−0.39	−0.41	−0.34	−0.92	0.03	0.03	0.15	0.14	0.36	−0.19
	M	−0.19	−0.64	−0.17	−0.54	0.12	−0.02	0.26	−0.07	0.36	0.00	0.80	0.72
	H	−0.29	−0.39	−0.42	−0.57	−0.54	−0.19	0.16	0.16	0.12	−0.03	0.12	0.53
200	E	−0.12	0.15	−0.08	0.15	−0.05	0.70	0.09	0.43	0.17	0.43	0.26	1.04
	M	−0.14	−0.05	−0.23	−0.05	−0.28	−0.65	0.09	0.24	0.02	0.23	0.04	−0.29
	H	−0.03	0.00	−0.11	−0.07	−0.02	0.36	0.18	0.28	0.14	0.20	0.29	0.70
500	E	−0.09	0.02	−0.04	−0.07	−0.17	0.32	0.00	0.13	0.07	0.04	−0.03	0.47
	M	−0.05	−0.05	−0.16	−0.02	−0.02	0.08	0.04	0.06	−0.05	0.09	0.11	0.23
	H	−0.11	−0.09	−0.11	−0.11	−0.06	−0.01	−0.02	−0.02	−0.01	−0.01	0.07	0.12
1000	E	−0.08	−0.13	0.01	−0.12	0.09	0.07	−0.04	−0.07	0.06	−0.07	0.15	0.14
	M	0.00	−0.10	−0.07	0.10	0.02	−0.11	0.04	−0.04	−0.02	0.15	0.08	−0.04
	H	−0.07	−0.03	−0.05	−0.04	−0.11	0.10	−0.02	0.02	0.00	0.01	−0.05	0.17
k = 10													
50	E	−0.57	−0.82	−0.35	−0.56	0.05	0.32	0.49	0.50	0.84	0.71	1.63	1.35
	M	−0.71	−0.55	−0.85	−0.76	−1.38	−0.60	0.34	0.76	0.31	0.51	0.13	1.09
	H	−0.50	−0.56	−0.64	−0.59	−0.78	−0.24	0.50	0.69	0.52	0.68	0.69	1.30
100	E	−0.39	−0.50	−0.33	−0.57	−0.94	−0.74	0.13	0.13	0.23	0.05	−0.21	0.05
	M	−0.52	−0.51	−0.43	−0.38	−0.81	−0.24	−0.01	0.13	0.15	0.24	−0.08	0.57
	H	−0.23	−0.34	−0.20	−0.41	−0.36	−0.86	0.28	0.30	0.38	0.21	0.39	−0.03
200	E	−0.16	−0.14	−0.16	−0.17	−0.05	0.20	0.09	0.18	0.12	0.14	0.31	0.59
	M	−0.13	−0.49	−0.18	−0.29	−0.32	−0.85	0.12	−0.17	0.09	0.02	0.04	−0.47
	H	−0.15	−0.39	−0.28	−0.22	−0.40	−0.48	0.10	−0.07	−0.01	0.09	−0.05	−0.10
500	E	−0.06	−0.01	−0.06	−0.02	−0.16	−0.12	0.04	0.12	0.05	0.11	−0.01	0.04
	M	−0.02	−0.08	−0.09	−0.18	−0.01	−0.26	0.09	0.05	0.02	−0.05	0.14	−0.09
	H	−0.09	−0.15	−0.08	−0.15	−0.14	−0.38	0.01	−0.01	0.04	−0.03	0.01	−0.22
100	E	−0.02	−0.03	0.02	−0.02	0.09	0.31	0.03	0.03	0.08	0.04	0.16	0.39
	M	0.02	0.05	−0.03	0.03	0.06	0.11	0.07	0.12	0.02	0.09	0.13	0.19
	H	−0.03	−0.02	−0.06	−0.07	−0.05	−0.28	0.02	0.05	−0.01	−0.01	0.02	−0.20
k = 15													
50	E	−0.61	−0.69	−0.57	−0.84	−1.04	−1.82	0.46	0.68	0.64	0.46	0.54	−0.13
	M	−0.78	−0.84	−0.72	−1.14	−0.90	−1.44	0.29	0.52	0.49	0.19	0.62	0.25
	H	−0.63	−0.65	−0.70	−0.43	−1.21	−1.03	0.46	0.71	0.52	0.91	0.35	0.65
100	E	−0.17	−0.17	−0.30	−0.15	−0.37	0.20	0.35	0.49	0.28	0.49	0.39	1.04
	M	−0.31	−0.33	−0.26	−0.46	−0.44	−0.33	0.22	0.34	0.33	0.19	0.34	0.51
	H	−0.21	−0.13	−0.37	−0.28	−0.17	0.56	0.31	0.53	0.22	0.35	0.59	1.39
200	E	−0.14	−0.20	−0.21	−0.25	−0.11	−0.35	0.11	0.12	0.08	0.05	0.26	0.04
	M	−0.18	−0.27	−0.16	−0.18	−0.32	−0.51	0.08	0.06	0.13	0.14	0.06	−0.11
	H	−0.09	−0.07	−0.15	−0.17	−0.03	0.31	0.17	0.27	0.14	0.15	0.35	0.73
500	E	−0.11	−0.21	−0.16	−0.13	−0.18	−0.39	−0.01	−0.08	−0.04	0.00	−0.04	−0.23
	M	−0.10	−0.20	−0.08	−0.20	0.05	−0.28	0.01	−0.06	0.03	−0.07	0.20	−0.11
	H	−0.06	−0.03	−0.02	−0.10	−0.04	0.04	0.04	0.10	0.10	0.03	0.11	0.20
1000	E	−0.01	−0.05	−0.08	−0.05	−0.11	−0.18	0.04	0.02	−0.02	0.01	−0.03	−0.09
	M	−0.02	−0.04	−0.03	−0.02	−0.09	−0.06	0.04	0.03	0.03	0.05	−0.01	0.02
	H	−0.02	−0.03	−0.02	−0.07	−0.06	−0.10	0.03	0.04	0.04	0.00	0.01	−0.02

All of the notations are described in Table 1

Table 3 illustrates the empirical power of homogeneity tests under various combinations of *k*, $\bar{n}$ and inequality of the sample sizes within each study for 20% and 50% heterogeneity of population matrices. Broadly speaking, there was increase in the power of homogeneity tests approximately in all scenarios of sample size designs when the number of MA studies and the sample sizes within each study were increased irrespective of the method studied. With a heterogeneity percentage equal to 20%, the power of the tests are ranked as MGLS ≥ UNIr ≥ TSSEM ≥ UNIz in all cases except for k = 5 and $\bar{n} = 50$ with equal and moderately unequal sized studies. Based on the results of this table, substantial reduction occurred in the power in moderately and highly unbalanced studies. By comparing moderately unequal and equal samples, the average rates of reduction of approximately 19, 17 and 13% were detected in the power of UNIr method, when the number of studies was equal to 5, 10 and 15, respectively. In UNIz approach, the reductions were approximately 23, 32 and 27% when *k* was equal to 5, 10 and 15, respectively. These rates were also about 17, 8 and 13% for the MGLS method for *k* = 5, 10 and 15. Moreover, there was reduction in the power of the TSSEM approach approximately by 21, 24 and 22% for the given value of *k*, respectively. For highly unequal sample sizes, more decrease of the power was obtained in comparison with equal sample sizes for each of the four methods than moderately unequal samples. There was an approximate decrease in the power of test by 36, 24 and 25% for the UNIr, 58, 50 and 47% for UNIz, 31, 17 and 22% for MGLS, and 54, 42 and 38% for TSSEM methods, for the same sequence of *k*.

**Table 3:** Rejection frequency percentage (statistical power) of stage 1 in heterogeneous studies

k	$\bar{n}$	Types	Low heterogeneity (20%)				High heterogeneity (50%)			
			UNIr	UNIz	MGLS	TSSEM	UNIr	UNIz	MGLS	TSSEM
5										
	50	E	40.1	37.6	44.6	47.6	39.0	35.3	40.4	43.9
		M	34.6	37.0	36.2	45.8	39.3	25.4	46.1	33.5
		H	22.9	11.3	29.6	18.4	38.6	20.0	45.8	25.6
	100	E	61.4	51.8	65.1	58.6	76.4	74.1	80.4	81.2
		M	46.3	31.1	52.0	37.1	71.1	58.1	78.1	65.0
		H	40.6	23.5	44.8	26.2	60.0	38.1	69.9	46.7
	200	E	93.6	90.6	96.3	92.9	98.5	98.3	99.4	99.4
		M	77.3	64.9	84.8	72.0	97.6	93.6	98.5	96.8
		H	63.0	45.7	69.6	50.6	89.7	79.9	93.0	83.6
	500	E	100	100	100	100	100	100	100	100
		M	99.5	99.3	100	100	100	100	100	100
		H	96.9	93.2	98.7	95.2	99.9	99.9	100	100
10										
	50	E	45.0	36.6	47.2	44.4	57.8	59.3	59.6	67.7
		M	34.7	19.3	42.3	28.0	67.6	48.0	76.0	60.9
		H	32.5	14.4	40.0	23.0	63.0	39.9	76.7	57.5
	100	E	82.9	75.3	84.2	78.2	94.7	94.9	96.9	97.6
		M	63.9	47.1	73.9	56.1	93.6	86.1	96.3	92.1
		H	56.1	29.4	62.7	37.4	91.4	81.7	96.3	88.1
	200	E	99.5	99.0	99.6	99.3	100	100	100	100
		M	95.5	88.3	97.7	91.2	100	100	100	100
		H	86.4	69.7	90.3	72.9	99.8	99.5	100	99.9
	500	E	100	100	100	100	100	100	100	100
		M	100	100	100	100	100	100	100	100
		H	100	99.5	100	99.9	100	100	100	100
15										
	50	E	56.8	42.7	59.4	55.9	72.5	72.6	76.0	81.5
		M	45.5	25.1	47.2	36.0	75.8	53.6	79.0	68.9
		H	35.9	14.1	39.9	27.9	73.3	40.8	77.0	59.4
	100	E	94.0	88.3	95.0	90.7	99.0	99.3	99.4	99.5
		M	77.3	57.8	78.7	65.3	98.4	95.5	98.9	96.4
		H	63.8	37.4	65.5	44.7	96.8	88.9	97.7	92.9
	200	E	100	100	100	99.9	100	100	100	100
		M	98.7	94.8	99.2	97.4	100	100	100	100
		H	94.4	82.7	96.4	85.4	100	100	100	99.9
	500	E	100	100	100	100	100	100	100	100
		M	100	100	100	100	100	100	100	100
		H	100	100	100	100	100	100	100	100

$\bar{n}$ average sample sizes, *k* number of studies, *UNIr* univariate-r, *UNIz* univariate-z, MGLS modified generalized least squared, *TSSEM* two-stage structural equation modeling, *E* equally sized studies, *M* moderately unequal sized studies, *H* highly unequal sized studies

When the heterogeneity of correlation matrices was 50%, the same results were observed, except for the TSSEM method in which the power of the test was to be relatively higher than the others when the sample sizes were equal. Moreover, less decrease was observed in this condition for the average of the power compared to 20% heterogeneity under different unequal sample sizes designs. It should be noted that these results were obtained when the average sample sizes were less than 500. When the sample size was equal to or greater than 500, the power was approximately similar for all methods and no substantial reduction was observed.

### Results of stage 2

Table 4 summarizes the results of Chi square test statistics and their standard deviations for evaluation of model fit of four methods in all conditions of sample sizes. In general, the true model significantly was over-rejected by UNIr and UNIz methods. The difference between the observed and expected values of Chi square statistics was increased significantly when the $\bar{n}$ and k increased. The lowest and the highest positive bias referred to moderately unequal and highly unequal samples of UNIr method when k = 5, $\bar{n} = 50$ and k = 15, $\bar{n} = 1000$, respectively. However the test statistics of MGLS and TSSEM approaches tended to converge to the expected means and standard deviation in almost all conditions. Furthermore, there was no dramatic difference for moderately and highly unequal than equal sample sizes for all approaches.

**Table 4:** Chi square statistics and their standard deviations of stage 2

$\bar{n}$	Types	k = 5				k = 10				k = 1			
		UNIr	UNIz	MGLS	TSSEM	UNIr	UNIz	MGLS	TSSEM	UNIr	UNIz	MGLS	TSSEM
50	E	2.3 (3.9)^b^	2.4 (4.0)^b^	1.0 (1.6)	1.1 (1.6)	2.5 (3.3)^b^	2.6 (3.4)^b^	1.1 (1.6)	1.1 (1.7)^a^	2.7 (3.7)^b^	2.8 (3.7)^b^	1.1 (1.7)	1.2 (1.8)^b^
	M	2.1 (2.8)^b^	2.2 (2.9)^b^	1.0 (1.4)	1.1 (1.5)	2.3 (3.2)^b^	2.3 (3.3)^b^	1.0 (1.4)	1.1 (1.4)	2.7 (3.7)^b^	2.7 (3.7)^b^	1.0 (1.4)	1.0 (1.4)
	H	2.3 (3.26)^b^	2.3 (3.4)^b^	1.0 (1.5)	1.0 (1.6)	2.4 (3.3)^b^	2.5 (3.4)^b^	1.0 (1.4)	1.0 (1.4)	2.5 (3.5)^b^	2.5 (3.6)^b^	1.0 (1.4)	1.0 (1.5)
100	E	2.3 (3.1)^b^	2.3 (3.1)^b^	1.0 (1.5)	1.0 (1.5)	2.9 (3.8)^b^	2.9 (3.9)^b^	1.1 (1.6)	1.1 (1.6)	3.4 (4.4)^b^	3.4 (4.4)^b^	1.1 (1.5)	1.1 (1.5)^a^
	M	2.4 (3.6)^b^	2.4 (3.6)^b^	1.0 (1.5)	1.0 (1.5)	2.8 (3.7)^b^	2.8 (3.7)^b^	1.0 (1.4)	1.0 (1.4)	3.4 (4.2)^b^	3.3 (4.2)^b^	1.0 (1.5)	1.0 (1.5)
	H	2.4 (3.2)^b^	2.4 (3.3)^b^	1.0 (1.5)	1.0 (1.5)	2.8 (3.7)^b^	2.8 (3.6)^b^	1.0 (1.4)	1.0 (1.4)	3.2 (4.0)^b^	3.2 (4.0)^b^	1.0 (1.4)	1.0 (1.4)
200	E	2.8 (3.7)^b^	2.8 (3.7)^b^	1.0 (1.3)	1.0 (1.3)	3.9 (4.6)^b^	3.9 (4.6)^b^	1.1 (1.44)	1.1 (14)	5.1 (5.9)^b^	5.1 (5.9)^b^	1.1 (1.6)	1.1 (1.6)
	M	2.7 (3.6)^b^	2.7 (3.7)^b^	1.0 (1.6)	1.1 (1.6)	3.6 (4.3)^b^	3.6 (4.3)^b^	1.0 (1.3)	0.9 (1.3)	4.8 (5.3)^b^	4.7 (5.3)^b^	1.1 (1.5)	1.1 (1.5)
	H	2.8 (3.6)^b^	2.9 (3.6)^b^	1.1 (1.6)^a^	1.1 (1.6)^a^	3.7 (4.6)^b^	3.7 (4.6)^b^	1.0 (1.4)	1.0 (1.4)	5.0 (5.55)^b^	4.9 (5.5)^b^	1.0 (1.4)	1.0 (1.4)
500	E	4.1 (4.9)^b^	4.1 (4.9)^b^	1.0 (1.4)	1.0 (1.4)	6.4 (6.2)^b^	6.4 (6.2)^b^	0.9 (1.3)	1.0 (1.3)	9.2 (7.79)^b^	9.1 (7.8)^b^	1.0 (1.5)	1.0 (1.5)
	M	4.1 (4.9)^b^	4.1 (4.9)^b^	1.0 (1.3)	1.0 (1.3)	6.8 (6.5)^b^	6.7 (6.5)^b^	0.9 (1.3)^a^	0.9 (1.3)^a^	9.8 (8.1)^b^	9.7 (8.0)^b^	1.0 (1.4)	1.0 (1.4)
	H	4.2 (4.9)^b^	4.2 (4.9)^b^	1.0 (1.5)	1.0 (1.5)	6.8 (6.4)^b^	6.7 (6.4)^b^	0.9 (1.3)	1.0 (1.3)	9.2 (7.8)^b^	9.1 (7.8)^b^	1.0 (1.4)	1.0 (1.4)
1000	E	7.0 (6.8)^b^	7.0 (6.8)^b^	1.1 (1.5)	1.1 (1.5)	11.7 (9.0)^b^	11.7 (9.0)^b^	1.0 (1.4)	1.0 (1.4)	15.9 (10.5)^b^	15.8 (10.5)^b^	1.0 (1.4)	1.0 (1.4)
	M	6.9 (6.4)^b^	6.9 (6.4)^b^	1.0 (1.4)	1.0 (1.4)	11.4 (8.7)^b^	11.3 (8.7)^b^	0.9 (1.3)	0.9 (1.3)^a^	15.9 (10.6)^b^	15.9 (10.6)^b^	1.1 (1.6)	1.1 (1.6)
	H	6.6 (6.5)^b^	6.6 (6.5)^b^	0.9 (1.3)	1.0 (1.3)	11.7 (9.0)^b^	11.7 (9.0)^b^	1.0 (1.4)	1.0 (1.4)	16.4 (10.8)^b^	16.3 (10.8)^b^	1.0 (1.4)	1.0 (1.4)

Values enclosed in parentheses represent standard deviations (SD). Expected means and SDs of Chi squares are 1 and 1.41, respectively

^a^p < 0.05; ^b^ p < 0.01

**Fig. 2:**
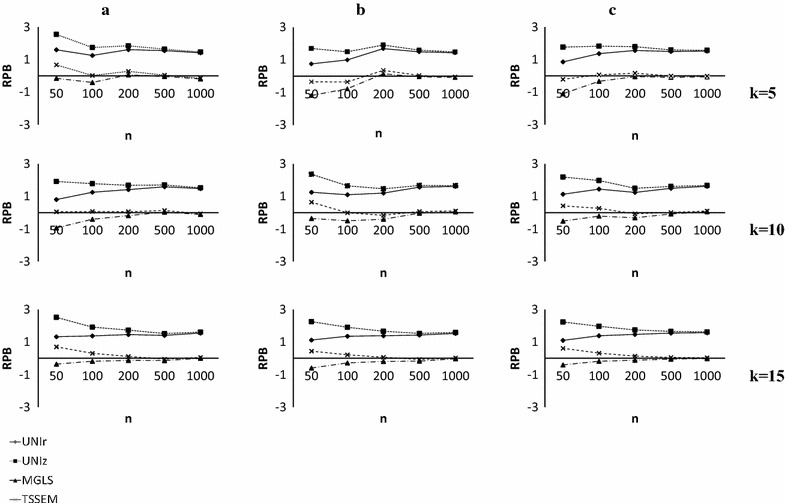
Relative percentage biases of parameters estimate in stage 2 for $\gamma_{11}$. *RPB* relative percentage biases, *n* average sample sizes, *k* number of studies, **a** Equally sized studies, **b** Moderately unequal sample sizes, **c** Highly unequal sample sizes

Figure 2 displays the relative percentage bias of parameter estimates for given values of *k*. Figure 2a–c shows the bias values of parameter estimates for the studies with equal, moderately unequal and highly unequal samples, respectively. As a result of the space limitations, one representative parameter, ${{\upgamma }}_{11}$, was selected to be displayed. Interested readers should refer to Additional file 1 for more details.

The results showed that the estimates of the four parameters (e.g., $\gamma_{11} ,\beta_{21} , \varphi_{12} \,{\text{and}} \,\psi_{22}$) were unbiased for UNIr and UNIz approaches with the values being lower than 5% in all studies. Two parameters, namely $\gamma_{12}$ and $\psi_{11}$, were close to 5% for almost all conditions. The lowest and highest values of relative percentage bias for the last parameter, $\gamma_{21}$, were 11.3 and 14.2%. However, for MMs, the relative unbiased estimates were observed for all the parameters in all combinations of the studies, inequality in the sample sizes and $\bar{n}$. In general, similar results were observed for the bias of the parameter estimates using the MGLS and TSSEM approaches. The relative percentage bias of the parameter estimates from these two methods was lower than 2% (the highest value was 1.97% for ψ_11 in TSSEM when k = 5, $\bar{n} = 50$ for study samples of the same size). Relative biases were attenuated slightly towards zero when $\bar{n}$ were increased.

**Fig. 3:**
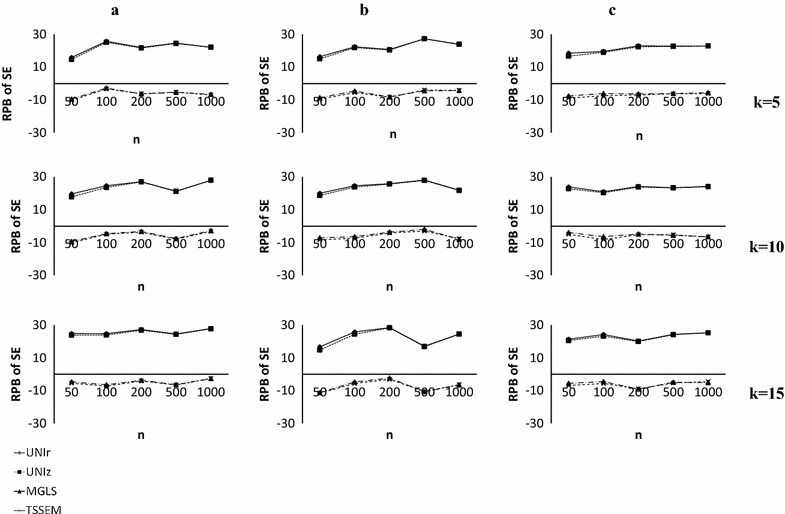
Relative percentage biases of the standard error of parameter estimates for $\gamma_{11}$. *RPB of SE* relative percentage biases of the standard error of parameter estimates for $\gamma_{11}$, *n* average sample sizes, *k* number of studies, **a** Equally sized studies, **b** Moderately unequal sample sizes, **c** Highly unequal sample sizes

Figure 3 compares the relative percentage biases of the standard errors (SE) of ${{\upgamma }}_{11}$ as one of the parameters of interest under different combination of sample sizes (Fig. 3a–c). Additional file 2 presents the rest of the parameter estimates in more detail. Using 10% as a good estimation of the relative biases, three SE of $\gamma_{11} , \gamma_{21} \;{\text{and}}\,\psi_{11}$ had relative biases larger than 10% for UMs, especially in small $\bar{n}$. The bias values for these parameters ranged from 13 to 29%. In almost all situations, there were positive biases for a larger number of parameters (three path coefficients and the factor correlation were positively biased). Moreover, the same pattern was observed for the bias values when the average sample sizes or the number of studies were increased. However, unlike the UMs, the results were different for MMs which were unbiased in almost all parameters, except one (e.g., $\gamma_{12}$, with the highest value being about 25% for TSSEM method). The relative percentage bias for these parameters ranged from 0 to 10.7%, 0 to 11.6%, and 0 to 14% in study sample sizes that were equal, moderately unequal, and highly unequal, respectively. These results showed that MGLS and TSSEM techniques had a similar performance. In these approaches, the relative percentage biases almost had a decreasing pattern when $\bar{n}$ increased. Slight negative biases were observed for three path coefficients ($\gamma_{11} , \;\beta_{21} \,{\text{and }}\gamma_{21}$), two error variance, $\psi_{11} \;{\text{and }}\psi_{22}$ and the covariance of observed X, $\varphi_{12}$. Generally, MMs outperformed the UMs in producing unbiased results for the parameters and their SE estimates. The relatively similar results were observed for all sample sizes designs.

## Discussion

This study examined the effect of unbalanced sample sizes designs in different primary studies on synthesizing MA methods in the first and second stages of MASEM. For a number of reasons, unequal sample sizes in different studies in MA and the centers in multicenter clinical trials commonly occur [30]. That is an issue, which has not yet been investigated, in the most previous simulation studies.

The present findings demonstrated that UM methods performed well in controlling Type I error rate for a combination of sample sizes and the number of MA except for a limited number of conditions. When the average sample sizes were lower than 200, MM methods, especially TSSEM, with moderately and highly unbalanced samples performed worse than UMs in the incorrect rejection of a true null hypothesis. However, when the average sample sizes were 200 or more, both UM and MM methods were closed to their nominal Type I error rates. These findings were in line with those generally reported by the researchers [13, 14] and Zhang for MM approaches [17]. These results imply that it is permissible to use any of the methods to estimate pooled correlation matrices in the first stage when there are relatively large sample sizes in the MA.

As compared with equal sample sizes designs, there was a decrease in the power of the UM and MM approaches for detecting heterogeneous studies when the same total sample size was assigned unequally. It is worth mentioning that as compared with moderately unequal sample sizes, studies with high inequality had more adverse effects on the power of homogeneity tests. Although the TSSEM approach provided a good balance between Type I error control and the statistical power in equal sample sizes design in this study and other published studies [13, 17], the present findings showed the relatively poor performance of this method for unequal sample sizes, especially in the $\bar{n}$ lower than 200, with highly unequal sample sizes. The results of this study showed that TSSEM had the highest power of rejecting the incorrect null hypothesis only when there was high heterogeneity in correlation matrices and the inequality of the sample sizes was negligible. Moreover, these results did not reveal the superiority of TSSEM method compared to other methods because there were inflation of the Type I error rates at the same points. However, the MGLS method had a high power for detecting heterogeneous correlation matrices regardless of the sample sizes and inequality used in the simulations. The obtained result is in agreement with the previous studies which had reported the good performance of MGLS approach [15, 17, 31].

Whether small studies are more heterogeneous than larger ones [32], the heterogeneity of correlation matrices were allocated to the small simulation studies. In addition, also, some other studies were considered as heterogeneous cases. Based on the present findings, MGLS and UNIr have more stability than UNIz and TSSEM methods even if the larger studies are selected as heterogeneous. In general, of the four tests of heterogeneity, MGLS and UNIr approaches have a higher statistical power in detecting heterogeneous studies than the two other methods. These findings are inconsistent with those of Cheung, who reported the superiority of TSSEM and unmodified-GLS procedures than the UM approaches [13].

The performance of UNIr and UNIz methods in Chi square test statistics to fit SEM was poor compared to MGLS and TSSEM approaches at the second stage. As shown by previous studies [11, 13], this test statistic had no good performance for UM approaches because it was affected by many factors, such as sample size [13]. In addition, when the number of studies increased, the Type I error rate related with the model fit exceeds the nominal level; therefore, the rate of such error increases. Generally, final decisions in SEM analyses cannot be achieved solely based on Chi square test, and many researchers have recommended utilizing a range of other goodness-of-fit indices to assess model fit [33]. Bollen demonstrated that the means of sampling distributions of Tucker-Lewis (TLI) and incremental fit (IFI) indices had relatively been unaffected by the sample size [34]. In the current study, the performance of some fit indices such as TLI and IFI were also assessed; but details of the results are not presented here. The results indicated good fit with negligible differences between the MM and UM methods. Further studies are required to assess the performance of combining correlation matrices approaches in more complex models, in fitting SEM at the second stage.

Based on the relative percentage bias of the parameter estimates and their SEs in the second stage, the present findings showed that MM approaches outperformed the UM approaches in almost all conditions. MMs produced fewer biased estimates of parameters and the SEs than UMs. These findings are consistent with those of Cheung and Chan [13] and Furlow et al. [11], in which they reported good performance of MM approaches in estimating the parameters and their SEs. It should be pointed out that the number of studies (**k**) included in the MA did not affect the estimation of the pooled correlation matrix in the first stage [35] or the biases of the parameters and the SE estimates in the second stage [11, 13, 15]. This is also true when considering the impact of unequal sample sizes in MA studies. However, when the total sample sizes increased, the biases of the parameter estimates decreased and also there was a reduction in the magnitude of the SEs but with a fluctuated pattern.

In the second stage of UM approaches, researchers choose different sample sizes, including arithmetic, weighted or total sample sizes. In the current study, based on the rule presented by Bollen, the total sample size was used to reduce the adverse effect of the sample sizes on SE of the parameter [36]. Nevertheless, UM approaches failed to yield satisfactory results. In general, using MM approaches for fitting SEM model in the second stage avoided the problems encountered using UM approaches, such as over-rejection of Chi square test, the goodness of fit indices, the power of homogeneity tests, and the relative biases of standard error of parameters [13]. Moreover, since it was difficult to consider the appropriate sample size in this stage for UM approaches; it seemed that MM approaches would be better choices for the analysis of MASEM in the second stage. However, owing to the popularity and ease of use for the users, many researchers still use UM approaches for the analysis of synthesized correlation matrices. UM approaches have good performance in controlling Type I error rates. Moreover, the relative percentage bias of the pooled correlation matrices is very good in the first stage, even under small or substantial unequal sample sizes. So it seems that, based on the current and other studies [13, 16, 23], there is no difficulty for applied researchers to use UMs in estimating pooled correlation matrices.

The present study had two main limitations which should be noted. First, comparison of the MA approaches with unbalanced sample sizes was performed under the fixed-effects model. In this model, the effect sizes of all studies in the MA are limited to one population effect size and the generalization of the results to main population is not possible [21]. However, many applied researchers use fixed-effects models in the MASEM studies [11]. Secondly, the estimation of the pooled correlation matrix was based on the full observation with no missing variable in this simulation study. Cheung and Chan pointed out that when the more studies are included in MASEM, it will be more likely to have missing variables and heterogeneous correlation matrices in the MA studies [13]. In the present study, the value of 15 was considered as the largest number of studies in this simulation with no missing variable. It is suggested that further studies are necessary to assess the larger number of MA studies using random-effects models with missing correlations in the first and second stages of MASEM.

## Conclusion

In summary, MGLS was the most appealing approach in terms of Type I error rate, detecting heterogeneous studies and precision of parameter estimates under equal and unequal sample size designs. For large and balance sample sizes, the TSSEM can be applied not only in combining the correlation matrices, but also in estimating the parameters in the second stage. However, it is recommended that the UNIr and UNIz methods are only used for synthesizing the correlation matrices in the first stage.

## Additional files

**Additional file 1.** Relative percentage biases of parameter estimates in the path model at stage 2. The table shows the bias values of parameter estimates for the studies with equal, moderately unequal and highly unequal samples in the path model. $\bar{\varvec{n}}$ Average sample sizes **k** number of studies **UNIr** univariate-r **UNIz** univariate-z **MGLS** modified generalized least squared **TSSEM** two-stage structural equation modeling.

**Additional file 2.** Relative percentage biases of the standard error of parameter estimates in the path model at stage 2. The table shows the bias values of the standard errors of parameter estimates for the studies with equal, moderately unequal and highly unequal samples in the path model. $\bar{\varvec{n}}$ Average sample sizes **k** number of studies **UNIr** univariate-r **UNIz** univariate-z **MGLS** modified generalized least squared **TSSEM** two-stage structural equation modeling.

## Abbreviations

MA: meta-analysis
SEM: structural equation modeling
MASEM: meta-analytic structural equation modeling
UM: univatiate meta-analysis
MM: multivariate meta-analysis
UNIr: univariate-r
UNIz: univariate-z
MGLS: modified generalized least square
TSSEM: two-stage structural equation modeling
BA1: Bonferroni-adjusted at-least-one
CFA: confirmatory Factor Analysis
ML: maximum likelihood
ADF: asymptotically distributions free
SE: standard errors
