# Supplementary material for: A comparative study of the impacts of unbalanced sample sizes on the four synthesized methods of meta-analytic structural equation modeling
Source: BMC Res Notes. 2017 Sep 6;10:446. doi: 10.1186/s13104-017-2768-5 (PMC5585956; doi:10.1186/s13104-017-2768-5)
Supplement: Supplementary file 1 — Additional file 1. Relative percentage biases of parameter estimates in the path model at stage 2. The table shows the bias values of parameter estimates for the studies with equal, moderately unequal and highly unequal samples in the path model. $\bar{\varvec{n}}$n¯ Average sample sizes k number of studies UNIr univariate-r UNIz univariate-z MGLS modified generalized least squared TSSEM two-stage structural equation modeling. [file 13104_2017_2768_MOESM1_ESM.pdf]

Additional file 1 relative percentage bias of parameter estimates in the path model at stage 2 (Equal sized studies)

$\bar{n}$	Methods	k=5							k=10							k=15						
		γ_{11}	γ_{12}	β_{21}	γ_{21}	φ_{12}	ψ_{11}	ψ_{22}	γ_{11}	γ_{12}	β_{21}	γ_{21}	φ_{12}	ψ_{11}	ψ_{22}	γ_{11}	γ_{12}	β_{21}	γ_{21}	φ_{12}	ψ_{11}	ψ_{22}
50	UNIr	1.60	-7.29	-3.33	11.31	-0.86	5.81	-4.12	0.81	-6.69	-4.40	13.56	-0.51	6.30	-3.68	1.33	-6.85	-4.40	13.07	-1.95	6.46	-3.20
	UNIz	2.55	-6.45	-2.71	11.99	0.69	3.95	-5.75	1.91	-5.74	-3.66	14.24	1.27	4.15	-5.53	2.52	-5.87	-3.65	13.71	-0.10	4.20	-5.07
	MGLS	-0.15	-0.41	0.63	-1.47	0.32	-0.10	-0.42	-0.94	0.04	-0.34	0.24	0.10	0.35	0.17	-0.35	-0.16	-0.29	-0.53	-1.54	0.50	0.67
	TSSEM	0.67	0.46	1.33	-0.97	1.82	-1.97	-1.96	0.06	1.00	0.48	0.79	1.78	-1.78	-1.62	0.70	0.81	0.53	0.04	0.16	-1.68	-1.14
100	UNIr	1.25	-6.52	-4.18	13.07	-1.12	5.88	-3.70	1.26	-6.71	-4.26	13.27	-0.84	6.29	-3.49	1.38	-6.42	-3.97	12.82	0.14	5.83	-3.57
	UNIz	1.74	-6.08	-3.88	13.41	-0.34	4.91	-4.52	1.78	-6.23	-3.86	13.55	0.01	5.24	-4.40	1.92	-5.93	-3.60	13.15	1.06	4.74	-4.50
	MGLS	-0.41	-0.02	-0.22	0.14	-0.53	0.14	0.02	-0.40	-0.09	-0.15	-0.37	-0.54	0.35	0.39	-0.18	-0.28	-0.12	0.05	0.34	0.31	0.05
	TSSEM	0.03	0.42	0.13	0.39	0.20	-0.81	-0.76	0.08	0.36	0.27	-0.10	0.25	-0.66	-0.50	0.31	0.19	0.28	0.35	1.18	-0.75	-0.84
200	UNIr	1.61	-6.21	-4.06	13.07	0.60	5.26	-3.81	1.41	-6.45	-4.09	13.19	0.15	5.88	-3.63	1.46	-6.45	-4.03	13.10	-0.38	5.97	-3.59
	UNIz	1.84	-6.00	-3.93	13.22	0.98	4.79	-4.18	1.68	-6.21	-3.92	13.35	0.58	5.35	-4.07	1.74	-6.22	-3.85	13.27	0.07	5.44	-4.05
	MGLS	0.06	-0.11	-0.25	0.50	0.89	-0.21	-0.21	-0.18	-0.13	-0.11	-0.04	0.30	0.17	0.11	-0.12	-0.13	-0.02	-0.23	-0.28	0.24	0.17
	TSSEM	0.28	0.09	-0.10	0.65	1.24	-0.67	-0.58	0.06	0.09	0.08	0.11	0.70	-0.33	-0.31	0.12	0.11	0.18	-0.10	0.11	-0.28	-0.26
500	UNIr	1.55	-6.38	-4.09	13.31	0.28	5.70	-3.74	1.59	-6.25	-4.04	13.25	-0.14	5.65	-3.70	1.41	-6.31	-4.03	13.21	-0.40	5.90	-3.61
	UNIz	1.64	-6.29	-4.03	13.37	0.44	5.52	-3.90	1.70	-6.16	-3.97	13.31	0.03	5.44	-3.88	1.52	-6.21	-3.96	13.28	-0.22	5.69	-3.79
	MGLS	-0.04	-0.05	-0.07	-0.03	0.40	-0.03	0.04	0.04	-0.03	-0.08	0.07	-0.09	-0.01	0.03	-0.14	-0.09	-0.08	0.02	-0.37	0.25	0.13
	TSSEM	0.04	0.03	-0.01	0.03	0.55	-0.22	-0.11	0.13	0.06	0.00	0.12	0.08	-0.21	-0.14	-0.04	0.01	0.01	0.08	-0.20	0.04	-0.05
1000	UNIr	1.42	-6.38	-4.12	13.55	0.05	5.88	-3.77	1.48	-6.31	-4.04	13.43	0.30	5.76	-3.80	1.56	-6.27	-3.94	13.16	-0.18	5.74	-3.73
	UNIz	1.47	-6.33	-4.09	13.58	0.12	5.79	-3.85	1.53	-6.27	-4.01	13.47	0.38	5.66	-3.88	1.61	-6.23	-3.90	13.20	-0.09	5.63	-3.82
	MGLS	-0.19	0.06	-0.03	-0.07	0.11	0.04	0.09	-0.11	0.01	-0.01	0.00	0.33	0.01	0.00	0.00	-0.06	0.03	-0.07	-0.16	0.08	0.01
	TSSEM	-0.15	0.10	0.01	-0.04	-1.70	-2.08	0.60	-0.07	0.05	0.03	0.03	0.41	-0.08	-0.08	0.05	-0.01	0.07	-0.04	-0.07	-0.03	-0.08

Additional file 1 relative percentage bias of parameter estimates in the path model at stage 2 (Moderately unequal sized studies)

$\bar{n}$	Methods	k=5							k=10							k=15						
		γ_{11}	γ_{12}	β_{21}	γ_{21}	φ_{12}	ψ_{11}	ψ_{22}	γ_{11}	γ_{12}	β_{21}	γ_{21}	φ_{12}	ψ_{11}	ψ_{22}	γ_{11}	γ_{12}	β_{21}	γ_{21}	φ_{12}	ψ_{11}	ψ_{22}
50	UNIr	0.75	-7.15	-3.78	13.01	-1.18	6.38	-4.51	1.26	-6.95	-4.27	12.24	-0.80	6.27	-3.02	1.12	-7.19	-4.54	12.98	-1.58	6.92	-2.94
	UNIz	1.70	-6.29	-3.13	13.63	0.38	4.51	-6.15	2.37	-5.98	-3.50	12.83	1.01	4.09	-4.85	2.26	-6.16	-3.81	13.73	0.23	4.67	-4.83
	MGLS	-1.19	0.35	0.57	-0.96	0.05	-0.08	-0.50	-0.35	-0.62	-0.47	-0.29	-0.22	0.71	0.60	-0.59	-0.35	-0.36	-0.91	-1.19	0.84	1.05
	TSSEM	-0.34	1.21	1.28	-0.43	1.42	-1.94	-2.08	0.64	0.31	0.34	0.29	1.48	-1.38	-1.19	0.44	0.63	0.44	-0.27	0.50	-1.34	-0.78
100	UNIr	1.00	-6.76	-3.90	13.35	-0.22	6.19	-4.21	1.10	-6.55	-4.40	13.19	-0.34	6.18	-3.26	1.36	-6.67	-4.21	13.23	-0.40	6.20	-3.50
	UNIz	1.50	-6.35	-3.62	13.68	0.57	5.24	-5.00	1.65	-6.07	-4.04	13.52	0.52	5.11	-4.15	1.91	-6.18	-3.85	13.57	0.52	5.10	-4.42
	MGLS	-0.77	0.14	0.29	-0.45	0.35	0.09	-0.24	-0.49	-0.27	-0.52	0.29	-0.06	0.56	0.44	-0.28	-0.13	-0.11	-0.39	-0.20	0.31	0.37
	TSSEM	-0.35	0.57	0.64	-0.22	1.10	-0.84	-0.99	0.00	0.19	-0.13	0.58	0.76	-0.47	-0.42	0.22	0.34	0.28	-0.07	0.64	-0.76	-0.52
200	UNIr	1.69	-6.22	-3.95	12.83	-0.75	5.42	-3.74	1.21	-6.37	-3.97	13.32	-0.90	6.12	-3.77	1.39	-6.33	-4.12	13.33	-0.55	5.92	-3.60
	UNIz	1.91	-6.00	-3.78	12.97	-0.36	4.95	-4.14	1.47	-6.14	-3.79	13.47	-0.46	5.59	-4.21	1.66	-6.09	-3.93	13.48	-0.10	5.37	-4.06
	MGLS	0.14	-0.07	-0.12	0.17	-0.47	-0.10	-0.11	-0.40	0.04	0.07	-0.13	-0.76	0.32	0.05	-0.19	-0.03	-0.14	0.06	-0.45	0.21	0.16
	TSSEM	0.35	0.12	0.05	0.31	-0.11	-0.55	-0.50	-0.16	0.27	0.25	0.02	-0.38	-0.19	-0.37	0.07	0.20	0.06	0.21	-0.04	-0.31	-0.29
500	UNIr	1.50	-6.28	-3.92	12.99	0.04	5.67	-3.72	1.57	-6.40	-3.93	13.15	-0.28	5.85	-3.76	1.43	-6.40	-4.06	13.37	-0.29	5.98	-3.68
	UNIz	1.59	-6.20	-3.86	13.06	0.20	5.48	-3.88	1.68	-6.31	-3.85	13.22	-0.11	5.64	-3.94	1.54	-6.31	-3.98	13.43	-0.11	5.76	-3.87
	MGLS	-0.04	-0.15	-0.04	0.08	0.15	0.12	-0.05	-0.02	-0.04	0.12	-0.34	0.23	0.07	0.05	-0.17	-0.01	0.01	-0.19	-0.25	0.17	0.15
	TSSEM	0.04	-0.06	0.03	0.13	0.30	-0.07	-0.21	0.07	0.05	0.20	-0.28	-0.06	-0.13	-0.12	-0.07	0.08	0.09	-0.14	-0.08	-0.04	-0.02
1000	UNIr	1.44	-6.11	-3.91	13.19	-0.13	5.61	-3.80	1.61	-6.24	-3.93	13.16	0.10	5.61	-3.78	1.54	-6.25	-3.99	13.30	-0.07	5.72	-3.76
	UNIz	1.49	-6.07	-3.88	13.22	-0.05	5.52	-3.88	1.66	-6.20	-3.90	13.20	0.18	5.51	-3.87	1.59	-6.20	-3.96	13.34	0.02	5.60	-3.85
	MGLS	-0.08	-0.03	-0.05	0.32	-0.07	0.08	-0.15	0.06	-0.05	0.02	0.00	0.12	-0.02	-0.06	-0.03	-0.01	-0.01	0.02	-0.05	0.03	0.00
	TSSEM	-0.04	0.01	-0.01	0.34	-0.01	-0.01	-0.22	0.10	-0.01	0.06	0.03	0.20	-0.11	-0.14	0.02	0.04	0.03	0.05	0.04	-0.08	-0.09

Additional file 1 relative percentage bias of parameter estimates in the path model at stage 2 (Highly unequal sized studies)

$\bar{n}$	Methods	k=5							k=10							k=15						
		γ_{11}	γ_{12}	β_{21}	γ_{21}	φ_{12}	ψ_{11}	ψ_{22}	γ_{11}	γ_{12}	β_{21}	γ_{21}	φ_{12}	ψ_{11}	ψ_{22}	γ_{11}	γ_{12}	β_{21}	γ_{21}	φ_{12}	ψ_{11}	ψ_{22}
50	UNIr	0.85	-7.32	-3.99	12.80	-0.13	6.35	-4.20	1.13	-6.78	-4.13	12.56	-0.44	6.15	-3.45	1.10	-6.49	-4.33	12.70	-1.16	6.14	-3.07
	UNIz	1.76	-6.38	-3.31	13.41	1.42	4.42	-5.85	2.20	-5.78	-3.44	13.27	1.29	3.99	-5.23	2.23	-5.47	-3.55	13.37	0.66	3.88	-4.97
	MGLS	-1.09	0.01	0.27	-0.89	1.04	0.06	-0.21	-0.52	-0.39	-0.24	-0.15	0.13	0.54	0.21	-0.41	-0.45	-0.61	0.43	-0.78	0.75	0.45
	TSSEM	-0.21	0.86	0.97	-0.25	2.49	-1.81	-1.85	0.42	0.57	0.49	0.53	1.69	-1.52	-1.53	0.62	0.55	0.21	1.08	0.91	-1.45	-1.42
100	UNIr	1.37	-6.83	-3.91	12.66	-0.38	6.00	-3.80	1.44	-6.54	-4.10	13.25	-0.95	5.99	-3.73	1.39	-6.63	-4.00	12.69	0.48	5.99	-3.45
	UNIz	1.83	-6.41	-3.61	13.02	0.40	5.08	-4.62	1.98	-6.07	-3.74	13.58	-0.07	4.93	-4.63	1.97	-6.15	-3.62	13.01	1.40	4.88	-4.37
	MGLS	-0.33	-0.20	0.12	-0.57	0.18	0.17	0.00	-0.21	0.05	0.00	-0.33	-0.66	0.07	0.14	-0.18	-0.42	-0.10	-0.24	0.68	0.42	0.20
	TSSEM	0.06	0.25	0.47	-0.32	0.90	-0.77	-0.77	0.27	0.51	0.40	-0.06	0.16	-0.96	-0.73	0.32	0.05	0.31	0.03	1.51	-0.64	-0.68
200	UNIr	1.56	-6.39	-3.87	12.97	0.26	5.55	-3.94	1.24	-6.36	-3.94	13.02	-0.53	6.02	-3.64	1.47	-6.48	-4.00	13.08	0.28	5.88	-3.66
	UNIz	1.79	-6.17	-3.71	13.10	0.64	5.08	-4.33	1.50	-6.13	-3.76	13.18	-0.11	5.50	-4.07	1.75	-6.23	-3.80	13.23	0.73	5.33	-4.13
	MGLS	-0.05	-0.06	0.08	-0.07	0.55	-0.12	-0.22	-0.32	-0.14	-0.02	0.01	-0.39	0.40	0.06	-0.12	-0.17	0.00	-0.21	0.38	0.17	0.10
	TSSEM	0.17	0.13	0.24	0.07	0.89	-0.57	-0.60	-0.07	0.08	0.16	0.15	0.00	-0.10	-0.36	0.14	0.06	0.20	-0.07	0.80	-0.36	-0.35
500	UNIr	1.49	-6.36	-4.06	13.23	-0.05	5.77	-3.70	1.50	-6.35	-4.05	13.34	-0.40	5.86	-3.70	1.55	-6.36	-4.07	13.38	0.02	5.79	-3.73
	UNIz	1.59	-6.28	-3.99	13.29	0.09	5.59	-3.85	1.61	-6.25	-3.98	13.40	-0.23	5.65	-3.88	1.66	-6.26	-4.00	13.44	0.20	5.57	-3.90
	MGLS	-0.09	-0.06	-0.07	-0.02	0.06	0.08	0.06	-0.08	0.00	-0.01	-0.12	-0.34	0.08	0.11	-0.05	0.00	-0.02	-0.13	0.06	0.01	0.09
	TSSEM	-0.01	0.02	0.00	0.03	0.20	-0.10	-0.09	0.01	0.10	0.06	-0.06	-0.18	-0.12	-0.06	0.05	0.09	0.06	-0.06	0.22	-0.19	-0.09
1000	UNIr	1.52	-6.31	-4.06	13.31	0.08	5.73	-3.71	1.62	-6.29	-4.00	13.22	-0.29	5.72	-3.71	1.57	-6.31	-4.02	13.34	-0.11	5.76	-3.75
	UNIz	1.57	-6.27	-4.02	13.34	0.16	5.64	-3.79	1.67	-6.25	-3.96	13.25	-0.20	5.62	-3.80	1.62	-6.26	-3.98	13.37	-0.02	5.65	3.84
	MGLS	-0.05	-0.04	-0.07	0.04	0.14	0.04	0.04	0.06	-0.03	0.00	-0.11	-0.26	0.01	0.05	-0.01	0.01	0.02	-0.11	-0.09	0.00	0.05
	TSSEM	-0.01	0.00	-0.03	0.06	0.21	-0.05	-3.37	0.10	0.02	0.04	-0.09	-0.18	-0.09	-0.03	0.04	0.05	0.06	-0.08	-0.01	-0.10	-0.04
