# Supplementary material for: A comparative study of the impacts of unbalanced sample sizes on the four synthesized methods of meta-analytic structural equation modeling
Source: BMC Res Notes. 2017 Sep 6;10:446. doi: 10.1186/s13104-017-2768-5 (PMC5585956; doi:10.1186/s13104-017-2768-5)
Supplement: Supplementary file 2 — Additional file 2. Relative percentage biases of the standard error of parameter estimates in the path model at stage 2. The table shows the bias values of the standard errors of parameter estimates for the studies with equal, moderately unequal and highly unequal samples in the path model. $\bar{\varvec{n}}$n¯ Average sample sizes k number of studies UNIr univariate-r UNIz univariate-z MGLS modified generalized least squared TSSEM two-stage structural equation modeling. [file 13104_2017_2768_MOESM2_ESM.pdf]

Additional file 2 relative percentage bias of the standard error of parameter estimates in the path model at stage 2 (Equal sized studies)

$\bar{n}$	Methods	k=5							k=10							k=15						
		γ_{11}	γ_{12}	β_{21}	γ_{21}	φ_{12}	ψ_{11}	ψ_{22}	γ_{11}	γ_{12}	β_{21}	γ_{21}	φ_{12}	ψ_{11}	ψ_{22}	γ_{11}	γ_{12}	β_{21}	γ_{21}	φ_{12}	ψ_{11}	ψ_{22}
50	UNIr	15.82	12.53	2.35	-18.41	6.91	-25.73	-5.30	19.60	-6.69	-4.40	13.56	6.86	-24.37	-8.83	24.85	8.27	-0.90	-18.02	2.51	-26.07	-3.37
	UNIz	14.66	11.54	1.13	-19.22	5.55	-27.34	-7.26	17.83	7.46	-0.72	-22.67	5.21	-26.17	-10.60	23.77	6.64	-1.50	-18.91	0.74	-28.24	-4.85
	MGLS	-9.48	22.71	-6.19	-3.82	0.20	-4.42	-1.25	-9.41	16.59	-10.74	-9.79	0.13	-2.70	-1.66	-4.57	24.54	-9.60	-8.58	-4.30	-3.71	-1.91
	TSSEM	-10.31	22.62	-7.19	-4.94	-1.34	-4.91	-1.99	-10.19	16.52	-12.04	-11.02	-1.95	-3.58	-2.57	-5.46	24.21	-10.87	-9.79	-5.55	-4.73	-2.24
100	UNIr	25.77	11.20	0.98	-19.39	6.25	-22.25	-2.68	24.68	9.59	0.67	-18.39	6.66	-24.67	-6.73	24.76	7.32	0.13	-20.06	5.72	-25.89	-6.19
	UNIz	25.17	10.93	1.11	-19.51	5.56	-23.08	-3.33	23.65	9.16	0.35	-19.04	5.95	-25.51	-7.22	23.86	7.23	-0.26	-20.87	4.76	-26.62	-7.17
	MGLS	-2.75	19.95	-6.85	-6.78	0.00	-3.30	0.56	-4.64	21.67	-9.32	-9.29	0.04	-5.21	-4.05	-6.46	20.34	-7.28	-8.55	-1.42	-0.09	-4.69
	TSSEM	-3.24	20.61	-6.68	-7.19	-2.01	-4.47	0.82	-4.86	21.10	-9.50	-9.70	-0.76	-4.63	-4.34	-7.29	18.88	-7.21	-8.38	-2.38	-0.37	-5.49
200	UNIr	22.03	12.22	-0.15	-20.69	7.23	-24.01	-4.18	27.08	8.40	2.55	-15.75	3.20	-25.91	-6.07	27.23	9.93	1.89	-18.16	5.19	-23.67	-6.11
	UNIz	21.70	12.24	-0.33	-20.55	6.87	-24.21	-4.29	27.00	8.26	2.52	-15.91	2.83	-26.36	-6.54	26.89	9.49	1.70	-18.28	4.78	-24.27	-6.41
	MGLS	-6.36	16.41	-7.11	-5.96	0.40	-2.90	-1.77	-3.22	21.83	-9.17	-9.02	-3.55	-4.43	-3.89	-3.64	19.63	-8.75	-8.54	-1.37	-4.06	-0.25
	TSSEM	-6.22	16.19	-7.03	-6.41	0.03	-3.19	-2.16	-3.66	22.08	-9.59	-9.28	-3.72	-4.00	-3.65	-4.05	19.92	-8.91	-9.24	-1.89	-4.05	-0.43
500	UNIr	24.68	15.89	1.41	-18.14	5.27	-24.16	-4.63	21.31	10.73	5.09	-17.92	4.15	-24.95	-1.09	24.49	13.67	-1.71	-21.41	5.31	-23.62	-4.76
	UNIz	24.39	15.64	1.22	-18.34	5.20	-24.57	-4.86	21.14	10.62	4.98	-17.99	4.00	-25.11	-1.30	24.32	13.40	-1.76	-21.39	5.13	-23.93	-4.89
	MGLS	-5.33	21.05	-10.41	-7.43	-1.64	-2.37	2.73	-7.57	21.29	-7.13	-9.36	-2.43	1.01	3.06	-6.38	18.63	-8.27	-4.84	-1.09	-0.94	-1.59
	TSSEM	-5.31	20.78	-10.86	-8.17	-1.83	-2.73	2.54	-8.04	20.98	-7.20	-9.09	-2.22	0.69	3.45	-6.59	18.71	-8.50	-5.04	-1.56	-1.19	-1.89
1000	UNIr	22.09	12.34	3.88	-17.97	4.62	-22.58	-5.98	27.88	10.67	2.68	-17.21	6.93	-23.56	-1.48	27.85	11.74	1.67	-18.10	6.54	-21.85	-3.43
	UNIz	22.08	12.25	3.92	-17.99	4.59	-22.66	-6.13	27.97	10.54	2.53	-17.26	6.85	-23.67	-1.71	27.70	11.61	1.68	-18.17	6.43	-21.95	-3.31
	MGLS	-6.63	21.85	-6.34	-6.66	-1.70	-2.08	0.60	-2.72	24.10	-7.26	-8.59	0.32	0.07	-0.44	-2.68	22.36	-8.18	-8.17	-0.24	-1.39	1.63
	TSSEM	-6.94	21.74	-6.22	-6.40	-2.27	-2.66	0.47	-3.18	24.39	-7.10	-8.59	0.04	-0.19	0.11	-2.60	23.18	-8.63	-8.05	-0.72	-0.80	2.47

Additional file 2 relative percentage bias of the standard error of parameter estimates in the path model at stage 2 (Moderately unequal sized studies)

$\bar{n}$	Methods	k=5							k=10							k=15						
		γ_{11}	γ_{12}	β_{21}	γ_{21}	φ_{12}	ψ_{11}	ψ_{22}	γ_{11}	γ_{12}	β_{21}	γ_{21}	φ_{12}	ψ_{11}	ψ_{22}	γ_{11}	γ_{12}	β_{21}	γ_{21}	φ_{12}	ψ_{11}	ψ_{22}
50	UNIr	16.36	9.94	0.84	-19.28	8.14	-23.51	-9.19	20.01	10.30	-0.34	-19.56	5.77	-25.69	-4.54	16.69	8.50	-1.47	-22.32	4.82	-26.43	-8.38
	UNIz	15.02	9.38	1.01	-19.99	6.58	-25.04	-11.03	18.73	8.75	-1.19	-20.17	4.23	-27.53	-5.49	14.71	7.34	-1.80	-22.76	3.62	-28.66	-9.65
	MGLS	-8.72	23.31	-9.29	-8.86	1.15	-1.46	-6.11	-7.10	24.21	-7.30	-6.09	-1.05	0.24	-3.00	-11.23	17.89	-9.30	-8.42	-1.20	-5.42	-4.82
	TSSEM	-9.59	24.39	-9.80	-8.64	-0.26	-3.56	-5.84	-8.37	23.79	-7.99	-7.75	-1.97	-0.51	-4.38	-11.55	18.13	-10.14	-9.87	-3.03	-5.80	-4.13
100	UNIr	22.38	7.45	-0.63	-19.50	4.67	-24.02	-5.70	24.66	9.12	0.96	-19.32	3.95	-24.46	-7.23	25.86	12.44	-1.27	-20.77	5.47	-22.33	-8.03
	UNIz	21.86	7.28	-0.92	-19.81	3.97	-24.74	-6.32	23.85	8.84	0.70	-19.49	3.25	-25.42	-7.80	24.41	11.93	-1.61	-21.19	4.67	-23.40	-8.95
	MGLS	-4.60	18.95	-10.34	-10.53	-2.02	-3.19	-2.17	-6.46	18.95	-10.52	-8.55	1.66	-2.32	-2.92	-4.66	17.34	-11.18	-8.16	-1.14	-1.72	-1.93
	TSSEM	-5.52	19.17	-10.29	-11.21	-2.57	-4.23	-2.83	-7.32	20.92	-11.26	-8.12	1.01	-2.85	-3.37	-5.59	16.71	-11.56	-8.58	-2.14	-2.52	-1.69
200	UNIr	20.79	13.05	2.88	-19.94	2.84	-24.04	-5.83	25.82	9.90	-1.06	-18.98	3.93	-20.91	-4.98	28.42	11.27	3.67	-15.34	5.39	-22.82	-4.52
	UNIz	20.43	12.85	2.66	-20.22	2.44	-24.43	-6.30	25.67	9.58	-1.03	-19.13	3.64	-21.52	-5.51	28.27	10.66	3.65	-15.40	4.96	-23.36	-5.22
	MGLS	-8.18	20.67	-6.47	-6.45	-2.86	-0.49	0.71	-3.68	21.70	-10.47	-9.78	-2.19	-2.95	-0.35	-2.33	24.44	-7.95	-6.65	0.00	-0.92	1.86
	TSSEM	-8.27	20.29	-6.63	-6.84	-3.65	-0.81	0.77	-4.21	21.75	-10.90	-10.11	-2.97	-0.48	-2.53	-3.08	23.46	-8.90	-7.05	-1.50	2.01	-0.89
500	UNIr	27.27	10.73	2.50	-20.18	10.41	-24.64	-2.91	28.08	11.22	0.64	-17.54	6.60	-20.93	-6.30	17.12	11.89	2.07	-19.80	2.01	-26.61	-2.87
	UNIz	27.23	10.56	2.46	-20.16	10.28	-24.82	-2.98	27.92	11.24	0.63	-17.67	6.45	-21.13	-6.40	16.86	11.79	1.97	-19.98	1.84	-26.89	-3.03
	MGLS	-4.40	18.51	-7.87	-8.70	3.91	-0.33	1.12	-1.93	23.00	-9.67	-7.28	0.01	-1.15	-1.84	-10.63	20.27	-6.31	-7.67	-4.23	-1.96	-0.84
	TSSEM	-3.99	19.15	-6.56	-8.94	3.31	-0.54	1.50	-2.95	22.19	-9.57	-7.81	-0.27	-1.22	-1.18	-10.48	21.18	-6.91	-6.96	-4.70	-2.16	-0.29
1000	UNIr	23.93	14.80	1.05	-18.16	8.67	-21.67	-3.47	21.90	16.46	0.81	-21.14	8.03	-23.71	-4.50	24.54	7.81	-2.11	-21.59	8.82	-24.48	-5.81
	UNIz	23.93	14.59	0.83	-18.30	8.62	-21.82	-3.69	21.77	16.34	0.71	-21.21	7.96	-23.86	-4.56	24.45	7.77	-2.21	-21.63	8.76	-24.58	-6.02
	MGLS	-4.29	20.79	-8.80	-7.92	1.66	-1.72	0.56	-8.02	16.77	-6.33	-3.02	0.59	-2.62	0.58	-6.56	16.47	-11.28	-11.11	1.55	-2.77	-2.69
	TSSEM	-4.17	20.29	-9.07	-8.37	1.62	-1.59	-0.57	-7.62	15.66	-5.66	-3.21	1.10	-3.54	1.21	-6.38	17.42	-11.16	-11.35	1.71	-3.15	-2.82

Additional file 2 relative percentage bias of the standard error of parameter estimates in the path model at stage 2 (Highly unequal sized studies)

$\bar{n}$	Methods	k=5							k=10							k=15						
		γ_{11}	γ_{12}	β_{21}	γ_{21}	φ_{12}	ψ_{11}	ψ_{22}	γ_{11}	γ_{12}	β_{21}	γ_{21}	φ_{12}	ψ_{11}	ψ_{22}	γ_{11}	γ_{12}	β_{21}	γ_{21}	φ_{12}	ψ_{11}	ψ_{22}
50	UNIr	18.54	10.83	-1.20	-19.88	14.11	-22.47	-10.42	23.92	7.79	-2.34	-20.26	3.99	-24.06	-7.35	21.35	9.39	-4.72	-20.48	3.66	-23.80	-7.34
	UNIz	16.67	9.55	-1.84	-20.95	12.80	-24.55	-11.36	22.84	6.68	-2.59	-20.65	2.16	-26.00	-8.64	20.60	7.95	-5.37	-20.97	1.90	-26.00	-8.58
	MGLS	-7.60	22.30	-10.45	-5.78	6.70	-1.12	-4.83	-3.84	19.83	-10.64	-10.10	-2.86	-4.43	-3.28	-5.56	18.83	-12.83	-8.85	-3.21	-3.48	-5.93
	TSSEM	-8.93	22.71	-10.31	-6.35	5.88	-2.03	-5.58	-4.96	19.40	-12.40	-11.42	-3.93	-5.73	-4.03	-6.87	19.63	-13.61	-10.47	-4.67	-6.56	-4.47
100	UNIr	19.54	10.83	0.71	-19.70	3.00	-22.57	-3.30	21.01	11.69	-0.28	-17.50	5.84	-24.46	-5.73	24.27	7.10	5.36	-16.15	3.94	-24.37	-4.90
	UNIz	18.97	10.28	0.42	-19.87	2.28	-23.4	-4.1	20.36	11.41	-0.98	-17.45	4.97	-25.43	-6.44	23.16	6.72	4.59	-16.96	3.21	-25.59	-5.64
	MGLS	-6.18	20.82	-10.25	-9.72	-3.80	1.02	2.62	-6.45	22.93	-10.93	-8.32	-1.06	-2.09	-2.64	-4.49	22.23	-8.88	-12.12	-3.37	-1.23	-1.81
	TSSEM	-7.41	21.59	-11.25	-10.15	-4.37	0.03	1.54	-8.15	24.77	-12.17	-8.80	-1.29	-2.36	-3.13	-5.66	21.98	-9.21	-12.77	-3.44	-1.71	-1.26
200	UNIr	23.02	8.56	1.51	-21.10	3.94	-23.93	-3.59	24.22	12.45	0.41	-19.45	4.42	-22.31	-2.79	20.29	10.70	-0.15	-20.63	4.72	-25.23	-11.28
	UNIz	22.41	8.09	0.85	-21.44	3.67	-24.51	-3.80	23.82	11.94	0.10	-19.67	4.05	-22.74	-3.24	19.91	10.31	-0.60	-20.88	4.26	-25.84	-11.71
	MGLS	-6.36	17.30	-5.61	-8.05	-2.37	-1.22	-1.32	-4.81	20.70	-5.96	-7.14	-2.63	-2.40	-1.78	-9.10	15.12	-13.28	-9.34	-1.76	-2.89	-2.85
	TSSEM	-6.97	17.28	-5.41	-8.65	-3.34	-1.30	-1.88	-5.21	20.38	-6.47	-7.58	-2.81	-2.29	-1.92	-8.90	14.78	-14.02	-10.28	-2.37	-2.90	-3.74
500	UNIr	22.78	15.22	-0.28	-19.05	8.75	-22.09	-2.97	23.36	12.09	2.69	-18.56	7.82	-23.94	0.71	24.35	11.74	5.62	-15.99	6.33	-21.45	-6.56
	UNIz	22.68	15.19	-0.59	-19.16	8.56	-22.27	-3.24	23.26	11.93	2.82	-18.62	7.59	-24.06	0.69	24.12	11.46	5.72	-16.24	6.19	-21.67	-6.86
	MGLS	-6.08	22.14	-8.40	-6.62	2.14	-0.77	-0.29	-5.64	21.43	-4.01	-6.73	1.07	-2.76	1.74	-4.94	24.09	-8.78	-7.38	0.23	-2.64	1.99
	TSSEM	-6.23	22.33	-8.83	-6.99	2.17	-0.94	-0.79	-5.27	21.52	-3.65	-6.46	0.72	-2.72	2.10	-5.27	23.77	-8.00	-7.58	-0.63	-2.96	2.10
1000	UNIr	22.93	11.23	3.13	-16.98	6.83	-25.92	-4.35	24.22	14.60	4.23	-18.21	5.62	-24.93	-7.83	25.33	12.89	5.18	-16.15	8.32	-19.93	-4.38
	UNIz	22.86	11.26	3.19	-16.97	6.79	-25.99	-4.31	24.04	14.50	4.22	-18.32	5.53	-25.08	-7.86	25.27	12.93	5.15	-16.23	8.25	-19.92	-4.45
	MGLS	-5.63	21.76	-4.76	-5.61	-0.42	-2.54	-3.48	-6.43	18.41	-9.52	-7.66	-0.33	-1.09	0.61	-5.08	23.98	-9.36	-8.15	1.77	3.10	4.84
	TSSEM	-6.12	21.66	-4.64	-5.47	-0.44	-2.78	-3.44	-6.64	18.74	-9.85	-7.25	-1.36	-2.47	0.79	-4.55	23.84	-8.57	-8.10	1.06	2.80	5.09
